# Religiosity and corruption tolerance in Muslim-majority countries

**DOI:** 10.3389/fsoc.2026.1858920

**Published:** 2026-09-03

**Authors:** Hussein Elkamel, Bayan Al Mahri

**Affiliations:** Public Law, Sultan Qaboos University, Muscat, Oman

**Keywords:** crime, criminological theories, institutional factors, Muslims, religiosity, tolerance for corruption

## Abstract

**Introduction:**

This study examines how religiosity shapes tolerance for corruption in societies where Muslims are the majority. It assesses how a person’s religious commitment influences attitudes toward corruption and compares this influence with criminological theories, including anomie, rational choice, amoral familism, and broken windows, which also explain tolerance for corruption.

**Methods:**

This study uses data from the World Values Survey (2017–2022) to identify factors influencing individuals’ tolerance toward corruption in Muslim-majority societies. The sample includes 5,050 respondents from 10 Muslim-majority countries. Using multiple regression models, the study seeks to explain corruption tolerance by employing criminological factors and religiosity to examine their relative explanatory power for the former.

**Results:**

The results suggest that the magnitude and significance of religiosity’s coefficients vary depending on how we measure religious commitments in Muslim-majority societies. In particular, criminological variables—particularly perceived detection risk and tolerance for minor offenses—appear to play a stronger, more consistent explanatory role in respondents’ tolerance toward corruption. The findings highlight the importance of institutional quality and enforcement mechanisms in shaping attitudes toward corruption, suggesting that strengthening legal accountability and governance structures may enhance the effectiveness of religious ethical norms in strengthening anti-corruption attitudes.

**Discussion:**

This research contributes to the literature on religion and tolerance for corruption by examining Muslim populations in countries where Islam is the dominant religion. It integrates religious and criminological explanations to examine how social norms, institutional conditions, and religious commitment interact to shape individuals’ tolerance for corruption.

## 1 Introduction

The relationship between religiosity and corruption in Muslim-majority countries is complex. Although corruption—including bribery, embezzlement, and abuse of power—is explicitly condemned in Islamic teachings (Ahmed, 2018; Alazzabi et al., 2020), many Muslim-majority countries continue to rank among the more corrupt countries globally^1^. This paradox raises a key question: Can religiosity shed light on tolerance towards corruption? Or are attitudes toward corruption better explained by broader criminological and institutional factors? And how do different measures of religiosity commitments in Muslim-majority countries contribute to individuals’ tolerance for corruption?

Religion is often viewed as shaping moral values and preventing corruption, but studies show the connection is complex, context-dependent, and sometimes unexpected. A consensus has yet to be reached, though some studies indicate that regions or countries with a strong religious heritage, particularly Protestantism, tend to experience lower levels of corruption. Conversely, some studies have suggested that hierarchical religious traditions may be associated with higher levels of corruption, particularly in contexts where religious authority is closely intertwined with political and institutional structures (Xu et al., 2017; Zelekha and Avnimelech, 2023; Paldam, 2001). Greater religiosity is sometimes linked to increased corruption, regardless of religious denomination (Gokcekus and Ekici, 2020; Zelekha and Avnimelech, 2023; Ali, 2025). Zelekha and Avnimelech (2023) show that hierarchical religions are associated with higher levels of corruption through their dominant-majority religious status, rather than the percentage of people belonging to a specific religion, thereby giving rise to the distinction between cultural and personal channels.

From a legal perspective, several studies have examined the underlying causes of corruption, such as ineffective law enforcement and fragile legal frameworks. The legal system’s success in shaping attitudes toward corruption is seen as a crucial factor in reducing the number of individuals engaging in corrupt practices, irrespective of their religion. Ultimately, tolerance for corruption highlights the pursuit of private gain as the primary motive. Criminological theories explain why individuals tolerate corruption; however, one challenge is that, in principle, Islamic teachings discourage criminal behavior, implying that higher levels of religiosity should be associated with less tolerance toward corruption. If criminological explanations accurately capture the motivations behind corruption, the perceived relationship between religion and corruption may be overstated when criminological and institutional factors are not adequately considered.

The study adopts the following criminological theories: Amoral familism theory, which posits that a strong connection to a mediating family member can override personal moral values, leading to greater tolerance for corruption. Anomie theory explains how individuals rationalize or neutralize their morals when their actions violate internal standards, increasing tolerance for corruption. The broken windows theory illustrates how minor offenses by individuals can promote greater tolerance for corruption. Finally, the Rational Choice theory suggests that people’s attitudes toward corruption depend on their calculations of the likelihood of getting caught. Overall, these criminological frameworks emphasize that understanding a person’s tolerance for corruption requires considering the quality of institutions and the social environment in which they operate.

To address these complexities, this study examines religiosity and criminological dimensions and factors in relation to individuals’ tolerance for corruption, using a comprehensive survey dataset from the 2017–2022 seventh wave of the World Values Survey. The dataset includes 5,050 respondents from 10 Muslim-majority countries. Although the sample comprises countries with Muslim majorities, creating a relatively homogeneous group, the countries differ in their legal systems. Most have adopted a mixed legal system framework, allowing varying degrees of interaction with Sharia law, except for Turkey, which maintains a fully secular judicial system (see Appendix Table 8). Additionally, Islamic Sharia law primarily governs family and personal status rather than anti-corruption policies.

The study uses five religiosity measures that the literature suggests affect individuals’ attitudes toward corruption: conceptual, practical, nominal, obedient, and affiliated religiosity. Regarding corruption, the study employs three measures of individuals’ tolerance for corruption: seeking government benefits to which they are not entitled, cheating on taxes, and giving or accepting bribes.

This paper offers several important contributions to the current body of research. First, it contributes to the literature by highlighting that most studies overlook criminological factors that influence an individual’s tolerance for corruption. Understanding these explanations is crucial because they are applicable regardless of a person’s religion. These factors are more than mere control variables; they directly affect individuals’ tolerance for corruption. Secondly, criminological theories depend on an individual’s perceptions of social norms, as well as their institutional and value beliefs, and these perceptions cannot be assumed to be consistent across different societies and individuals. Many Muslim-majority countries experience lower institutional quality and income levels, which can create moral and social risks that may be unobserved in studying the religion-corruption relationship. Thirdly, excluding criminological explanations might heighten the conflict between religious anti-corruption teachings and the persistent corruption in countries with religious majorities. Finally, religion’s effect on corruption is often questioned: whether the relationship is real or merely masked by social, cultural, or institutional contexts that link individuals to corruption. Thus, this paper explores different aspects of religiosity, including conceptual, practical, nominal, obedient, and affiliated forms, while considering various social, cultural, institutional, and demographic factors.

This study shows that criminological theories explaining why individuals tolerate corruption are more explanatory than religiosity alone in Muslim-majority countries. The results indicate that individuals’ religiosity contributes to lower tolerance for corruption, particularly through conceptual, practical, and obedience-related factors. Additionally, criminological frameworks such as rational choice and broken windows offer compelling explanations for tolerance of corruption, highlighting the critical roles of the rule of law, robust law enforcement, and improvements in institutional quality. In summary, these findings underscore the need to strengthen institutional accountability and uphold the rule of law to raise anti-corruption attitudes among Muslims.

The study is organized as follows: Section 2 presents a literature review of related studies. Section 3 details the data and methodology. Section 4 presents the results, Section 5 provides the discussion, and Section 6 concludes.

## 2 Literature review

### 2.1 Religiosity, criminology, and corruption

Islamic teachings strongly condemn corruption and emphasize justice, honesty, trustworthiness, and accountability as essential values for individuals and society. The Qur’an, Hadith, and Islamic jurisprudence (fiqh) explicitly prohibit acts such as bribery (risywah), embezzlement (ghulul), fraud (tadlis), and hoarding (ihtikar), treating them as serious violations that undermine social justice and public trust (Khoirin and Junaedi, 2022; Alazzabi et al., 2020; Din et al., 2023). In principle, these religious norms suggest that stronger religiosity should discourage corruption. Yet despite these explicit prohibitions, corruption remains widespread in many Muslim-majority countries. This apparent contradiction has prompted scholars to examine the gap between religious ideals and social practice. Previous studies emphasize integrating Islamic ethical principles into governance, education, and legal systems while addressing institutional and cultural barriers that may weaken their practical implementation (Salahudin et al., 2025; Zelekha and Avnimelech, 2023; Hudaib, 2020).

Empirical research, however, provides mixed evidence on the relationship between religion and corruption. Ko and Moon (2014) tested this relationship through a comparative analysis across multiple religions and four proposed linkages and found no statistically significant support for it. Other studies suggest that religiosity may reduce corruption by strengthening moral values and promoting ethical behavior. Still others find that the relationship is conditional on broader institutional and social factors. For example, Valdovinos-Hernandez et al. (2019) argue that most religions are not systematically associated with corruption and that earlier studies may have overstated the connection. Similarly, Sommer et al. (2012) find that religion’s impact on corruption depends on institutional conditions, particularly the level of democracy and religious freedom within a country. In contrast, Ali (2025), analyzing data from 52 countries, finds that religiosity may be positively associated with corruption and negatively related to sustainable development. These contrasting findings suggest that the relationship between religion and corruption cannot be understood solely through religious doctrine but must also account for broader institutional and societal contexts.

To better understand why corruption persists even in societies with strong religious norms, several scholars have incorporated criminological explanations into their analyses. Criminological theories focus on the motivations and social conditions that shape individuals’ attitudes toward corruption. Rational Choice Theory, for instance, holds that individuals’ tolerance for corruption rises when expected benefits outweigh perceived risks, particularly in environments with weak accountability and opportunities for misconduct (Graycar and Sidebottom, 2012; Zimring and Johnson, 2005; Suparman et al., 2024). In such contexts, corruption may occur regardless of individuals’ religious beliefs.

Other studies emphasize the role of social structures and cultural norms. Research on amoral familism shows that strong kinship networks and family-based loyalty can foster favoritism and nepotism, potentially undermining broader social cooperation and increasing tolerance for corruption (López and Santos, 2014; Bliznyuk, 2021; Catanzaro, 2018). Similarly, the concept of anomie highlights how weakened social norms and institutional instability can reduce moral regulation and increase acceptance of unethical behavior (Cruz, 2021; Ohu and Spitzmueller, 2023; Yarim and Çelik, 2021; Akhayeva and Turgunbayeva, 2023). In environments with low institutional trust and fragile governance, individuals may be more likely to rationalize corrupt practices (Santos et al., 2025).

Finally, the Broken Windows Theory suggests that tolerance for minor violations can create an environment where more serious forms of corruption become normalized. When small acts of misconduct go unpunished, they signal weak enforcement and reduce public confidence in institutions, potentially encouraging further unethical behavior (Keuschnigg and Wolbring, 2015; Schram et al., 2022). Theories of criminality explain why individuals’ attitudes toward corruption are crucial to religion-corruption studies, because these attitudes operate independently of religiosity. Additionally, these theories highlight the social and institutional structures of societies, which have been a focus of studies that question the impact of religiosity on corruption.

Despite these contributions, the literature has not reached a clear consensus on the relationship between religiosity and corruption. Much of the debate centers on religious affiliation or doctrinal teachings, while comparatively little attention has been paid to the interplay among religiosity, criminological factors, and institutional conditions. This study seeks to address this gap by examining how religiosity and criminological explanations jointly shape individuals’ tolerance of corruption in Muslim-majority societies.

### 2.2 Islamic jurisprudence perspectives on modern corruption

This study uses three proxies to assess tolerance for corruption, reflecting respondents’ attitudes toward it. However, it is important to consider the overlap and relationship between these proxies and the Islamic teachings respondents adhere to. Typically, Muslims aim to take actions that fall into one of three categories: to bring benefits (jālb al-masālīh), to prevent corruption (dar’ī al-mafāsid), or to verify whether a contract is valid or invalid (Ahmed, 2018).

Respondents’ answers to the first corruption proxy question, asking whether “claiming an unentitled government benefit” is justifiable, should adequately reflect attitudes toward corruption among religious Muslims, since such tolerance is forbidden by Islamic teachings (Ahmed, 2018). Public money is regarded as sacred, and any unjust appropriation is classified as ghulul (embezzlement), with strong warnings in the Qur’an and Sunnah for those who misuse public funds (Aripin, 2025). More generally, any government revenue collected that benefits someone else is considered a bribe (Alatas, 1989).

The second measure of corruption is “cheating on taxes,” which reflects respondents’ attitudes toward corrupt acts. The literature indicates that while Islam requires Muslims to pay zakat as a religious duty, it also recognizes the legitimacy of state taxes in certain situations—primarily when public needs exceed what zakat can meet (Fidiana, 2020; Gueydi, 2022). Tax evasion is widely regarded as unethical and haram (prohibited) because it undermines social justice and public welfare (Aliyu et al., 2016). However, tax evasion might not be considered immoral, especially if taxes are perceived as unfair or misappropriated by authorities (Abodher et al., 2020). Studies show that religiosity alone does not reliably prevent tax evasion; factors such as fairness, social norms, and perceptions of government legitimacy are crucial in shaping compliance (McGee, 2012; Emzaed, 2018; Abodher et al., 2020).

The final proxy for corruption, “accepting bribes,” directly indicates respondents’ attitudes towards corruption. Bribery is regarded as inherently and unquestionably haram because it undermines justice, deprives individuals of their rights, and encourages socio-political corruption (Jabbar, 2013; Ahmed, 2018). Prophetic traditions condemn all parties involved—givers, receivers, and intermediaries—and regard the acceptance of bribes as a grave sin (Narrated by Ahmed, Al-Bazaz, and Al-Tabarani). Some scholars (e.g., al-Nawawī, Yūsuf al-Qaradāwī) permit paying bribes in extreme cases to defend a right or prevent significant injustice when all lawful options are exhausted; however, the receiver still commits a sin (Roza et al., 2020; Faisol et al., 2023).

Overall, the three corruption measures we employ should be opposed by a Muslim with religious convictions, as these indicators clearly fall under actions forbidden by Islamic teachings, except for “cheating on taxes,” if the respondent perceives the government as unjust or prone to misuse of funds. Nonetheless, our control variables—reflecting the country’s institutional quality—should mitigate these effects. The paper proposes that Muslims’ attitudes toward corruption are shaped by multiple dimensions of religiosity, alongside institutional and criminological factors, and aims to explore the paradox that, despite Islamic teachings condemning corruption, many Muslim countries exhibit high levels of corruption.

## 3 Data and methodology

### 3.1 Data

This section draws on selected questions from the World Values Survey Wave 7 (2017–2022) to primarily test the effect of religiosity on corruption. The dataset covers 10 countries with Muslim-majority populations (*n* = 5,050), namely Bangladesh, Indonesia, Iran, Iraq, Libya, Malaysia, Nigeria, Pakistan, Tunisia, and Türkiye. Although Muslims also reside in countries where non-Muslims are the majority, we selected a sample of Muslims living in countries with Muslim majorities. Our goal is to focus on Muslim respondents living in countries where Islam is the dominant religion, allowing us to examine religiosity within a relatively homogeneous religious context (Zelekha and Avnimelech, 2023). Individual-level survey data have several advantages over country-level data, especially for addressing ecological inference issues. They provide direct measurements rather than expert perceptions, especially regarding corruption.

Moreover, the survey records not only tolerance for corruption but also diverse values related to socioeconomic, political, traditional, and normative factors. It also includes demographic details about respondents. Below, we introduce the dependent and independent variables. Respondents’ answers, such as Do not know, No answer, Not applicable, Missing, or Not available, may be assigned a specific negative value. These responses were removed, leaving only answers within the question’s scale. The WVS-7 questionnaires are available in English, Spanish, Arabic, and Russian. Additionally, in each country, the questionnaire must be translated into all languages spoken as a first language by at least 15% of the population. Our sample consists of more than 5,000 respondents across 10 Muslim countries, where language and cultural differences might influence respondents’ perceptions of the survey questions, potentially reflected in their answers and affecting our results. On the WVS website, measures taken during questionnaire development should mitigate this concern: although the survey questions were proposed and developed in English, the questionnaires were designed by social scientists from around the world. Moreover, the questionnaire was tested through independent back-translations into English to verify accuracy; for each translated version, the survey was independently translated back into English to check whether it matched the original survey. In addition, the translated version underwent pretesting to identify questions with problematic translations. Our methodology should further mitigate bias arising from these unobservable variations in respondents’ perceptions of the questionnaire, given that responses were collected in multiple languages.

#### 3.1.1 Dependent variable

Some studies rely on measures of corruption that draw on expert perceptions from international organizations such as Transparency International (TI CPI) (Zelekha and Avnimelech, 2023; Valdovinos-Hernandez et al., 2019). This study prefers a measure that links an individual’s religiosity to their attitudes toward corruption rather than to those of other international experts. Additionally, corrupt practitioners often struggle to admit their misconduct, even when they view themselves as victims. Therefore, following Ko and Moon (2014), we will select a dependent variable that assesses tolerance for corruption using WVS questions about attitudes toward three types of corrupt tolerance: “claiming government benefits to which you are not entitled,” “cheating on taxes,” and “someone accepting a bribe in the course of their duties.” These three variables are rated on a 1–10 scale, with 10 indicating that individuals always justify the underlying corrupt tolerance and 1 indicating that they never do. In other words, the higher (lower) the value, the greater (lesser) the respondent’s tolerance toward corruption.

Although these questions capture attitudes toward corruption rather than direct involvement in corrupt activities, tolerance for corruption is widely used in cross-national research as a proxy for individuals’ ethical acceptance of it. Previous studies using World Values Survey data show that attitudes toward the justifiability of corruption are strongly associated with broader societal corruption norms (Ko and Moon, 2014; Gokcekus and Ekici, 2020).

#### 3.1.2 Independent variables

Our independent variables are grouped into three categories: (1) variables measuring individuals’ religiosity, (2) variables measuring criminal tendencies, and (3) control variables accounting for differences across individuals and countries that contribute to individuals’ tolerance for corruption. Although our dataset captures country-level differences in institutional and criminality factors at both the country and individual levels, we note that country-level and individual perceptions of these factors are not measured on the same scales, given the heterogeneity among these countries’ legal systems (see Appendix Table 8).

First, the religiosity measures are located in the Religious Values section of the survey. This section includes six core questions that assess respondents’ religiosity across the conceptual, practical, nominal, obedience, and affiliation dimensions. Questions 169–170 address respondents’ conceptual beliefs. Question 169, on a 4-point scale, asks respondents which they prioritize when religion and science conflict. Question 170, also on a 4-point scale, asks respondents whether they consider their religion (Islam) the only acceptable religion. The practical questions, Q171 and Q172, focus on the frequency of prayer and attendance at religious services. These two questions capture religious commitments on 7- and 8-point scales, respectively. Self-religiosity is identified based on responses to Q173, in which respondents indicate their level of religiosity on a 3-point scale, with the lowest level being atheist. The frequency of prayer (Q172) serves as an indicator of behavioral religiosity, enabling the study to distinguish between nominal religious identification (Q173) and actual religious practice. Thus, Q169 represents conceptual religiosity, Q172 practical religiosity, and Q173 nominal religiosity.

Moreover, religious commitments may be captured through mediating factors such as *affiliation* and *obedience*, as Ko and Moon (2014) indicated. Question 94, on a 3-point scale, measures affiliation by asking respondents whether they are active, inactive, or not members of a religious organization. Obedience is calculated using four questions (Q8, Q14, Q15, and Q17) that highlight qualities to be promoted at home. These qualities include independence (Q8), determination (Q14), religious faith (Q15), and obedience (Q17)^2^.

Affiliation with religious communities may influence individuals’ perceptions of authority and accountability, potentially due to cultural and institutional factors. Moreover, followers of hierarchical religions often show obedience to authority, especially in countries where the state supports religion or where religious tension exists, and there is no clear separation between religion and government. This close connection between the state and religion fosters a more tolerant view of state abuses and correlates with increased corruption, especially among respondents who are religiously affiliated or who value obedience and religious faith as legitimate values to be taught to children, rather than independence and determinacy.

Assessing the validity of a measure of religiosity as an indicator of religious commitments is inherently challenging. For example, self-identification can be biased: an individual might claim to be religious out of affirmation, even if they are not, or deny being religious out of humility, even if they are. Additionally, someone might intellectually consider themselves religious but not truly follow religious commands, such as a commitment to intolerance of corruption. This potential for bias exists across all religious measures, so we aim to examine multiple aspects of religiosity to capture a broader picture of religious commitment. To address the paradox of religious individuals’ tolerance for corruption despite Islamic teachings condemning it, the best approach is to employ a range of religious measures to understand this complex issue. Overall, our religiosity variables are *conceptual*, *practical*, *nominal, obedience,* and *affiliation*^3^.

The second group of variables draws on criminological theories explaining why individuals might engage in corrupt activities. Those theories are universal and not related to respondents’ levels of religious commitment. The study employs four measures to capture respondents’ attitudes toward corruption, informed by criminological doctrine. First, amoral familism theory describes a social pattern in which loyalty to one’s immediate family takes precedence over broader social or moral duties, often leading to support for criminal acts. The survey’s Social Values section investigates people’s relationships with family, friends, and other groups. Q1 measures the importance of family in respondents’ lives on a 4-point scale from 1 (very important) to 4 (not at all important). However, we should note that this is an indirect measure; amoral familism suggests that individuals prioritize family interests over the public good and does not necessarily conflict with someone who values family.

Anomie theory emphasizes a breakdown in social cohesion (such as declining moral standards). Several questions in the Ethical Values and Norms section of the survey reflect the social breakdown component of Anomie. However, we selected Q176, which assesses how often the individual struggles to determine the correct moral rules to follow. The questions are rated on a scale from 1 to 10, where 1 means “agree entirely” and 10 means “completely disagree.”

The Broken Windows theory goes beyond mere damage; it symbolizes neglect and conveys an unspoken message that small offenses are unimportant in that environment. Therefore, we use a minor offense to assess whether respondents justify fare evasion on public transportation and whether this, in turn, leads to more tolerant attitudes toward corruption. Q178 is rated on a scale from 1 to 10, with 1 meaning never justifiable and 10 meaning always justifiable.

The final theory in criminology is Rational Choice, which holds that individuals’ attitudes toward corrupt practices are shaped by weighing the benefits against the potential risks. Question 120 in the survey’s corruption section effectively measures this perception. It asks whether the perceived risk of being held accountable for giving or receiving bribes, gifts, or favors in public service is very high in the country. The question uses a scale from 1 to 10, where 1 signifies no risk at all, and 10 indicates a very high risk.

Lastly, the study controls for several variables that capture differences among individuals and their home countries. Individual variables include gender (binary), age (in years), education (on an 8-point scale), and income (on a 10-point scale). Country differences are represented by three variables from the ICRG survey: socioeconomic condition, religious tension, and law & order; higher values indicate better performance. These variables are used to account for cross-country heterogeneity across our 10-country dataset. Notably, substantial variations in these factors may influence respondents’ tolerance of corrupt practices. Socioeconomic conditions are measured by unemployment, consumer confidence, and poverty, whereas law and order has two components: the strength and fairness of the legal system and the extent to which people obey the law.

Table 1 presents descriptive statistics for the 10 countries, with the final row showing the overall sample mean. Across all countries, respondents were generally less tolerant of accepting bribes but more open to claiming undeserved government benefits. Regarding religiosity, the conceptual, practical, and nominal indices indicated that respondents were quite religious, whereas obedience and affiliation were less reflective of their religiosity. In criminological theories, respondents valued family highly but rated themselves as moderate in their ability to determine the right moral rules. Additionally, they were less tolerant of justifying minor offenses and perceived a significantly higher risk of being held accountable for corruption.

**Table 1:** Descriptive statistics.

Country	Statistics	Corruption indices			Religiosity indices					Criminality			
		Q177	Q180	Q181	Q169	Q172	Q173	Obedi.	Q94	Q1	Q120	Q176	Q178
Bangladesh	Mean	1.90	1.66	1.56	1.22	6.46	2.97	0.6	0.53	1.02	6.25	4.84	1.65
	Medi	1	1	1	1	7	3	1	0	1	6	5	1
	Max	10	6	10	3	8	3	2	2	3	10	10	9
	Min	1	1	1	1	1	2	-2	0	1	1	1	1
Indonesia	Mean	2.90	2.176	1.766	1.62	7.73	2.89	0.457	1.285	1.013	8.2	4.867	2.63
	Medi	2	1	1	1	8	3	0	2	1	9	5	1
	Max	10	10	10	4	8	3	2	2	3	10	10	10
	Min	1	1	1	1	1	2	-2	0	1	1	1	1
Iran	Mean	3.63	2.40	1.50	2.11	6.80	2.84	−0.001	0.59	1.09	6.00	5.88	2.7
	Medi	3	1	1	2	8	3	0	0	1	6	6	1
	Max	10	10	10	4	8	3	2	2	4	10	10	10
	Min	1	1	1	1	1	1	−2	0	1	1	1	1
Iraq	Mean	3.89	2.70	2.79	1.29	7.53	2.76	0.83	0.28	1.03	8.15	7.10	3.24
	Medi	3	2	2	1	8	3	1	0	1	9	7	2
	Max	10	10	10	4	8	3	2	2	3	10	10	10
	Min	1	1	1	1	1	1	−2	0	1	1	1	1
Libya	Mean	2.99	1.401	1.26	1.15	7.83	2.77	0.77	0.99	1.01	5.06	5.66	1.59
	Medi	1	1	1	1	8	3	1	1	1	5	6	1
	Max	10	10	10	4	8	3	2	2	2	10	10	10
	Min	1	1	1	1	1	2	−2	0	1	1	1	1
Malaysia	Mean	4.49	3.42	3.11	1.44	7.61	2.88	0.18	0.58	1.01	7.37	4.50	3.98
	Medi	5	3	2	1	8	3	0	0	1	8	4	4
	Max	10	10	10	4	8	3	2	2	2	10	10	10
	Min	1	1	1	1	1	1	−2	0	1	1	1	1
Nigeria	Mean	1.90	1.72	1.57	1.41	7.82	2.94	0.88	1.22	1.01	5.80	4.95	1.96
	Medi	1	1	1	1	8	3	1	2	1	6	5	1
	Max	10	10	10	4	8	3	2	2	2	10	10	10
	Min	1	1	1	1	2	1	−2	0	1	1	1	1
Pakistan	Mean	2.56	1.91	1.51	1.26	7.28	2.93	0.62	0.33	1.11	6.75	5.21	1.79
	Medi	1	1	1	1	8	3	1	0	1	7	5	1
	Max	10	10	10	4	8	3	2	2	4	10	10	10
	Min	1	1	1	1	1	1	−2	0	1	1	1	1
Tunisia	Mean	2.88	1.77	1.61	1.31	6.49	2.60	0.31	0.08	1.03	6.48	3.23	1.95
	Medi	1	1	1	1	8	3	0	0	1	7	3	1
	Max	10	10	10	4	8	3	2	2	4	10	10	10
	Min	1	1	1	1	1	1	−2	0	1	1	1	1
Turkey	Mean	1.86	1.64	1.62	2.00	6.39	2.81	0.11	0.07	1.08	7.16	5.45	2
	Medi	1	1	1	2	7	3	0	0	1	7	5	1
	Max	9	10	10	4	8	3	2	2	3	10	10	10
	Min	1	1	1	1	1	1	−2	0	1	1	1	1
	S-mean	2.90	2.08	1.83	1.48	7.20	2.84	0.47	0.59	1.04	6.72	5.17	2.35

However, country-level descriptive statistics reveal considerable differences. For the corruption proxy “claiming government benefit,” respondents in Iran, Iraq, and Malaysia showed the highest tolerance for justifying such corrupt acts. Regarding the “cheating on taxes” and “accepting bribes” proxies, these countries also had the highest acceptance levels, except in Iran, where respondents were more inclined to justify accepting bribes. The lowest responses for all three proxies were observed in Turkey and Bangladesh. Responses indicating that accepting bribes could be justified occurred less frequently than those for “benefiting from unentitled government benefits” and “cheating on taxes.” Accepting bribes often led respondents to view such acts as unjustifiable, given the inherently corrupt nature of the act. Conversely, statements about tax evasion or claiming unentitled benefits might resonate more with respondents who feel vulnerable to inequality or perceive government injustice.

Regarding religiosity, the strongest belief is conceptual, with respondents in Bangladesh, Libya, and Pakistan reporting higher levels, whereas the lowest levels of conceptual religiosity are in Iran and Turkey; however, respondents in these countries still agree with prioritizing religion over science when the two conflict. Regarding practical religiosity, measured by the number of prayers per day, respondents in Libya and Nigeria reported the highest levels, while those in Turkey and Bangladesh reported the lowest among the countries surveyed. In terms of nominal religiosity, respondents in Tunisia identified as religious less frequently, whereas those in Bangladesh did so most often. Respondents in Iran and Turkey showed the lowest levels of promoting obedient and religiously faithful qualities, whereas respondents in Iraq and Nigeria reported the highest levels. When affiliation was measured as a form of religiosity, most respondents were either not members or inactive members of a religious organization, though respondents in Indonesia and Nigeria displayed the highest levels of religiosity in this respect. Clearly, religiosity levels varied among individuals and countries depending on the specific variable used to measure religiosity.

Finally, four criminology theories explain individuals’ attitudes toward corruption. Amoral familism suggests that most people consider family very important, with little variation across the countries in our sample. The second theory concerns difficulties in identifying the correct moral rules; respondents in Iraq and Iran showed the greatest ability to determine these rules, while those in Tunisia were, on average, the most likely to struggle. Most respondents across countries were moderate on this scale. The broken window theory indicated that respondents in Iraq and Malaysia were most tolerant of minor offenses such as skipping fares on public transport, whereas respondents in Bangladesh and Libya were least tolerant of such acts. The final indicator, based on rational choice theory, posits that individuals’ attitudes toward corruption depend on their perceived risk of being caught. Respondents in Indonesia, Iraq, and Malaysia believed there was a high chance of accountability for corruption, while those in Libya perceived the lowest risk. Interestingly, despite Iraq’s high levels of corruption, respondents there still perceived a high risk of being caught, which seems contradictory. However, because the study examined individual religiosity and tolerance for corruption rather than average levels, this distinction is important.

### 3.2 Methodology

Our primary analysis will rely on logit models because our dependent variables are ordinal. However, we first apply Ordinary Least Squares (OLS) regression to establish a baseline for analyzing the relationship between religion and corruption. However, it assumes equal distances between categories, which can lead to biased standard errors or predictions that fall outside category bounds. The benefits of using OLS here include straightforward calculations and easily interpretable coefficients. It also serves as a quick starting point for more advanced models like ordered logistic regression. OLS may perform adequately because our dependent variable, measured on a 1-to-10 scale, behaves almost like a continuous measure. Furthermore, our extensive sample size (*N* > 5,000) supports reliable hypothesis testing due to the Central Limit Theorem. Lastly, interpreting the results is straightforward: a one-unit increase in X corresponds directly to a clear change on the 1-to-10 scale. Overall, our goal is for the OLS coefficients to closely match the results from the ordered logit model.

Then, we employ logit regression as our main approach. Specifically, we use ordered-logit models as all the dependent variables are ordinal on a scale of 1 to 10 points. Unlike linear regression, these models do not assume equal spacing between categories. An ordered-choice model evaluates fit using Pseudo *R*-squared, as standard *R*-squared is not suitable for categorical outcomes. It excludes a constant term because the estimated cut-off points act as the intercept. The model sets this constant to zero and estimates cut points for identification. Ordered-choice models rely on z-statistics because parameters are estimated via Maximum Likelihood Estimation (MLE), which depends on asymptotic properties that converge to a standard normal distribution with larger samples.

The study proposes the following equation:


$$\begin{array}{c} \text{Corruptio}n_{i,j}=\alpha +b_{1}\text{Religio}n_{i,j}+b_{2}\text{AmoralFamilis}m_{i,j}\\ +b_{3}\text{Anomi}e_{i,j}+b_{4}\text{Brokenwindo}w_{i,j}\\ +b_{5}\text{Rationalchoic}e_{i,j}+b_{6}\text{gende}r_{i,j}\\ +b_{7}\mathrm{ag}e_{i,j}+b_{8}\text{educatio}n_{i,j}+b_{9}\text{incom}e_{i,j}\\ +b_{10}\text{SociEc}o_{j}+b_{11}\text{ReligTe}n_{j}\\ +b_{12}\text{LawOr}d_{j}+\varepsilon_{i,j} \end{array}$$ (1)

Corruption measures individuals’ tolerance for corrupt practices and serves as the dependent variable, with three alternative measures. As for the independent variables, “Religion” reflects religious traits spanning religiosity, conceptual, practical, nominal, affiliation, and obedience. Criminology encompasses theories such as Anomie, Amoral Familism, Broken Windows, and Rational Choice. Gender, age, education, and income are individual-level control variables, whereas *SociEco*, *ReligTen*, and *LawOrd* control for country differences. However, one limitation of the country control variables is that they take a single value for all respondents in the same country, so standard errors will be underestimated and z-statistics inflated; therefore, the coefficients on those variables should be interpreted with caution and in an associative sense.

## 4 Results

### 4.1 Main results

The study tests its hypothesis using Equation 1, which employs multiple regression and alternative measures of the core indices. The findings are presented in various tables, each highlighting a different corruption-tolerant variable, with both OLS and logistic regression analyses included. Table 2 shows that most measures of religiosity were not significant, except for obedience. This suggests that individuals who prioritize obedience are less likely to justify tolerance of corruption, such as claiming government benefits for which they do not qualify. Religiosity, whether conceptual, practical, nominal, or institutional, showed no significant effect.

**Table 2:** The role of religiosity and criminology on individual tolerance for corruption.

Ordered logit	[1]	[2]	[3]	[4]	[5]
Conceptual religiosity	−0.019				
	(−0.51)				
Practical religiosity		−0.0008			
		(−0.05)			
Nominal religiosity			−0.066		
			(−0.91)		
Obedient religiosity				−0.095***	
				**(−3.44)**	
Affiliated religiosity					0.030
					(0.89)
Amoral familism	−0.174	−0.179	−0.185	−0.199*	−0.173
	(−1.45)	(−1.49)	(−1.54)	**(−1.66)**	(−1.44)
Anomie theory	0.033***	0.033***	0.033***	0.032***	0.033***
	**(3.17)**	**(3.14)**	**(3.16)**	**(3.11)**	**(3.17)**
Broken window	0.464***	0.464***	0.463***	0.463***	0.463***
	**(33.33)**	**(33.29)**	**(33.27)**	**(33.27)**	**(33.26)**
Rational choice	−0.036***	−0.036***	−0.036***	−0.035***	−0.036***
	**(−3.54)**	**(−3.51)**	**(−3.52)**	**(−3.42)**	**(−3.54)**
Soci-econ	0.115***	0.115***	0.115***	0.109***	0.116***
	**(8.01)**	**(7.97)**	**(8.00)**	**(7.59)**	**(8.04)**
Religious tension	−0.054*	−0.052*	−0.054*	−0.050	−0.049
	**(−1.72)**	**(−1.67)**	**(−1.73)**	(−1.59)	(−1.55)
Law & order	0.055	0.054	0.052	0.040	0.053
	(1.55)	(1.47)	(1.43)	(1.08)	(1.46)
Gender	−0.004	−0.003	0.001	−0.005	−0.001
	(−0.07)	(−0.06)	(0.02)	(−0.09)	(−0.02)
Age	0.0001	0.0002	0.0003	0.0002	−0.0001
	(0.08)	(0.09)	(0.18)	(0.12)	(−0.05)
Education	0.012	0.012	0.011	0.012	0.011
	(0.78)	(0.75)	(0.69)	(0.76)	(0.72)
Income group	0.005	0.005	0.005	0.005	0.005
	(0.36)	(0.36)	(0.36)	(0.38)	(0.37)
Log-likelihood	−7595.57	−7595.7	−7595.29	−7589.76	−7595.31
LR statistics	1627.61	1627.34	1628.18	1639.23	1628.14
Pseudo-*R*^2^	0.10	0.09	0.09	0.10	0.09

OLS	[1]	[2]	[3]	[4]	[5]
Religiosity	0.107	0.036*	−0.051	−0.062*	0.079**
amoral familism	−0.288**	−0.291*	−0.317**	−0.326**	−0.299**
Anomie theory	0.039***	0.039***	0.038***	0.038***	0.038***
Broken Window	0.560***	0.560***	0.558***	0.558***	0.556***
Rational choice	−0.025**	−0.024**	−0.024**	−0.023**	−0.024**

The dependent variable is the benefit not entitled to. *, **, and *** indicate statistically significant 10%, 5%, and 1%, respectively. Z-statistics are in the parentheses. Bold numbers indicate statistically significant coefficients, with p-values at 1%, 5%, or 10%.

In contrast, the crime theories explaining tolerance of corruption appear effective, as shown in Table 2. The signs of the amoral familism coefficients align with expectations, indicating that when people value their family loyalty, they are more likely to justify corruption. However, only the results from model 5 were marginally significant. Contrary to expectation, all coefficients related to anomie were positive and statistically significant. Because higher values on this scale indicate less trouble deciding on correct moral rules, these findings suggest that individuals with greater moral certainty are actually more likely to justify claiming undeserved government benefits. Notably, coefficients from broken windows theory were highly significant, implying that individuals tolerant of minor offenses are more likely to justify undeserved government benefits. According to rational choice theory, all coefficients were significant and aligned with expectations: as the perceived risk of detecting corrupt practices increases, individuals are less likely to justify claiming benefits to which they are not entitled.

Country control variables were highly significant for the socioeconomic coefficients, suggesting that even in countries with better socioeconomic conditions- that have low unemployment, low poverty, and better consumer confidence- individuals may still be willing to claim benefits to which they are not entitled. Coefficients related to religious tension appear to capture cross-country variation, as they indicate that individuals in countries with high religious tension are more likely to justify claiming benefits to which they are not entitled. However, these coefficients were insignificant in models 4 and 5 when obedience and affiliation were included. Coefficients on Law & Order were also insignificant. Individual control variables remained insignificant across all models. The bottom of the table highlights the main coefficients for the religiosity and criminality indices; OLS results closely match the logistic results.

Table 3 mirrors the specifications of Table 2 but replaces the dependent variable with “cheating on taxes” to measure respondents’ inclination toward corrupt tolerance. The coefficients for conceptual and practical religiosity are significantly and negatively associated with respondents’ attitudes toward justifying tax cheating, whereas other religiosity measures are not significant. Among criminological theories, only the broken windows and rational choice models are significant and show expected signs: ignoring minor offenses and a lower risk perception increase tolerance for tax cheating. Country-specific factors such as socioeconomic status and religious tension remain significant, similar to Table 2, and the law & order variable is now significant, indicating that people in countries with enforced law and order are less likely to justify tax cheating. Regarding individual traits, higher education seems to be negatively associated with tolerance for tax evasion in some models. The OLS results at the bottom of Table 3 are highly comparable.

**Table 3:** The role of religiosity and criminology on individual tolerance for corruption.

Ordered logit	[1]	[2]	[3]	[4]	[5]
Conceptual religiosity	−0.093**				
	**(−2.17)**				
Practical religiosity		−0.090***			
		**(−5.14)**			
Nominal religiosity			0.054		
			(0.65)		
Obedient religiosity				−0.043	
				(−1.36)	
Affiliated religiosity					−0.050
					(−1.27)
Amoral familism	0.088	0.059	0.114	0.101	0.102
	(0.72)	(0.48)	(0.94)	(0.83)	(0.83)
Anomie theory	−0.001	−0.002	0.0003	0.0002	0.0001
	(−0.10)	(−0.16)	(0.03)	(0.01)	(0.01)
Broken window	0.565***	0.563***	0.567***	0.566***	0.567***
	**(34.59)**	**(34.53)**	**(34.65)**	**(34.64)**	**(34.69)**
Rational choice	−0.050***	−0.051***	−0.051***	−0.051***	−0.051***
	**(−4.21)**	**(−4.35)**	**(−4.39)**	**(−4.34)**	**(−4.35)**
Sociec	0.140***	0.150***	0.141***	0.140***	0.139***
	**(8.52)**	**(9.09)**	**(8.64)**	**(8.52)**	**(8.46)**
Religious tension	−0.066*	−0.087**	−0.071**	−0.072**	−0.079**
	**(−1.84)**	**(−2.43)**	**(−1.98)**	**(−2.02)**	**(−2.19)**
Law & order	−0.136***	−0.149***	−0.127***	−0.136***	−0.128***
	**(−3.27)**	**(−3.59)**	**(−3.08)**	**(−3.26)**	**(−3.10)**
Gender	−0.066	−0.043	−0.074	−0.070	−0.073
	(−1.05)	(−0.68)	(−1.16)	(−1.11)	(−1.15)
Age	−0.002	−0.0005	−0.002	−0.002	−0.002
	(−0.87)	(−0.22)	(−0.95)	(−0.88)	(−0.84)
Education	−0.033*	−0.023	−0.029	−0.031*	−0.030
	**(−1.78)**	(−1.24)	(−1.59)	**(−1.66)**	(−1.62)
Income group	0.006	0.002	0.006	0.006	0.006
	(0.37)	(0.15)	(0.39)	(0.39)	(0.38)
Log-likelihood	−5571.79	−5561.32	−5573.92	−5573.2	−5573.32
LR statistics	1851.09	1872.03	1846.84	1848.29	1848.04
Pseudo-*R*^2^	0.14	0.14	0.14	0.14	0.14

OLS	[1]	[2]	[3]	[4]	[5]
Religiosity	−0.086***	−0.046***	0.062	0.0008	−0.016
Amoral familism	0.062	0.054	0.087	0.082	0.079
Anomie theory	−0.004	−0.003	−0.002	−0.003	−0.003
Broken window	0.497***	0.496***	0.498***	0.498***	0.498***
Rational choice	−0.021***	−0.022***	−0.022***	−0.023***	−0.023***

The dependent variable is “cheating on taxes”. *, **, and *** indicate statistically significant 10%, 5%, and 1%, respectively. Z-statistics are in the parentheses. Bold numbers indicate statistically significant coefficients, with p-values at 1%, 5%, or 10%.

In Table 4, we utilize the corruption variable of “accepting bribes” as the dependent variable. Religiosity coefficients are highly significant for conceptual, practical, and obedient religiosity, consistent with the results in Table 3, whereas coefficients for nominal and affiliation are insignificant. Across all criminological models, coefficients were significant only in the broken windows and rational choice models; that is, ignoring minor offenses and lower perceived risk increased respondents’ justification for accepting bribes. Results across all models were similar; socioeconomic conditions (low unemployment, low poverty, and better economic conditions) and religious tensions had positive coefficients, suggesting that countries that perform better on those two dimensions may not mitigate individuals’ attitudes toward accepting corruption. Law and order were highly significant and indicated that stronger law enforcement helps individuals to be less tolerant of accepting bribes. Regarding individuals’ characteristics, education seems to be associated with a lower likelihood of justifying bribery, whereas higher income may not mitigate this attitude as in the earlier models shown in Tables 2, 3, criminological theories of broken windows and rational choice remain valid and legitimate across all model specifications and different religiosity variables in explaining respondents’ attitudes toward corruption. Additionally, the results of all OLS models in Tables 2, 3, 4 are consistent with those of the ordered logit models.

**Table 4:** The role of religiosity and criminology on individual tolerance for corruption.

Ordered logit	[1]	[2]	[3]	[4]	[5]
Conceptual religiosity	−0.117***				
	**(−2.57)**				
Practical religiosity		−0.082***			
		**(−4.35)**			
Nominal religiosity			−0.092		
			(−1.03)		
Obedient religiosity				−0.088***	
				**(−2.59)**	
Affiliated religiosity					−0.008
					(−0.19)
Amoral familism	0.036	0.012	0.056	0.048	0.062
	(0.26)	(0.09)	(0.41)	(0.35)	(0.46)
Anomie theory	0.009	0.008	0.011	0.011	0.011
	(0.73)	(0.68)	(0.85)	(0.87)	(0.86)
Broken window	0.446***	0.445***	0.446***	0.446***	0.447***
	**(28.89)**	**(28.87)**	**(28.90)**	**(28.91)**	**(28.92)**
Rational choice	−0.051***	−0.052***	−0.053***	−0.052***	−0.053***
	**(−3.98)**	**(−4.10)**	**(−4.14)**	**(−4.08)**	**(−4.15)**
Sociec	0.200***	0.210***	0.203***	0.200***	0.202***
	**(11.44)**	**(11.91)**	**(11.62)**	**(11.39)**	**(11.49)**
Religious tension	0.184***	0.161***	0.171***	0.176***	0.173***
	**(4.62)**	**(4.05)**	**(4.32)**	**(4.45)**	**(4.33)**
Law & order	−0.468***	−0.471***	−0.458***	−0.473***	−0.456***
	**(−10.27)**	**(−10.41)**	**(−10.14)**	**(−10.35)**	**(−10.07)**
Gender	−0.011	−0.007	−0.006	−0.015	−0.014
	(−0.16)	(−0.11)	(−0.09)	(−0.22)	(−0.21)
Age	−0.003	−0.001	−0.002	−0.003	−0.003
	(−1.21)	(−0.69)	(−1.09)	(−1.23)	(−1.21)
Education	−0.092***	−0.083***	−0.091***	−0.089***	−0.089***
	**(−4.58)**	**(−4.12)**	**(−4.53)**	**(−4.48)**	**(−4.46)**
Income group	0.038**	0.035**	0.039**	0.039**	0.039**
	**(2.26)**	**(2.08)**	**(2.31)**	**(2.32)**	**(2.31)**
Log-likelihood	−4859.57	−4853.7	−4862.31	−4859.48	−4862.83
LR statistics	1408.94	1420.68	1403.45	1409.12	1402.42
Pseudo-*R*^2^	0.13	0.13	0.13	0.13	0.13

OLS	[1]	[2]	[3]	[4]	[5]
Religiosity	−0.039	−0.028**	−0.047	−0.030	0.018
Amoral familism	0.044	0.035	0.049	0.046	0.056
Anomie theory	0.003	0.003	0.003	0.003	0.003
Broken window	0.370***	0.369***	0.370***	0.370***	0.370***
Rational choice	−0.018**	−0.018***	−0.018***	−0.018***	−0.018**

The dependent variable is “accepting bribes”. *, **, and *** indicate statistically significant 10%, 5%, and 1%, respectively. Z-statistics are in the parentheses. Bold numbers indicate statistically significant coefficients, with p-values at 1%, 5%, or 10%.

The odds ratio for religiosity and criminality, reported in the appendix, indicates that the broken-windows and rational-choice indicators are more consistently associated with corruption tolerance than the religiosity measures, with the broken-windows measure showing the strongest association. However, religiosity might still play a role in enhancing anti-corruption attitudes. Overall, our study’s religious indices seem to challenge the common belief that religion leads to increased corruption. Conversely, criminological theories are generally more aligned and consistently significant across various models and measures of tolerance for corruption. The overall fit of the model is quite satisfactory, with all log-likelihood estimates similar and consistently negative. The estimates for the dependent variable, accepting bribes, presented in Table 4, have the highest value, indicating the best fit among all tested models. The LR statistics across Tables 2–4 demonstrate that including all predictors significantly improves model performance. The highest Pseudo-R2 is 0.14, reflecting a moderate and acceptable fit for an ordered logit model. Although 0.14 seems low in ordinary least squares (OLS) regression, logistic regression evaluates fit differently; this value indicates that predictors add meaningful information compared to a model without predictors. The thresholds (cut-points) in the appendix show that all estimates increase monotonically, confirming the models’ mathematical validity and the consistency of the ordinal ranking across the latent scale.

### 4.2 Robustness test

Several criticisms challenge the assertion that religiosity influences tolerance for corruption (Ko and Moon, 2014), suggesting that broader sociological, normative, or cultural factors might be responsible rather than religiosity itself (Xu et al., 2017; Zelekha and Avnimelech, 2023). Therefore, we will re-estimate our primary regression specification as in Tables 2–4. After resampling our data and examining the effects on the coefficients for religiosity and criminality, we will not report coefficients for other variables due to space constraints. Resampling will address country- and respondent-level differences that may have contributed to the main results in the previous tables. The resampling is addressed in the following three sub-titles.

#### 4.2.1 Religiosity and corruption in the least religiously tense countries

One component of political risk in the ICRG is religious tension, which has a maximum score of 6 points; higher scores indicate lower risk, whereas lower scores indicate higher risk. This risk concerns a single religious group’s intent to replace civil law with religious law, exclude other religious groups from political influence, suppress religious freedoms, or seek dominance in governance. The tolerance for corruption among religious individuals can vary, and higher religious tension tends to correlate with increased rather than decreased tolerance for corruption. High levels of religious tension can be linked to the rise of strict hierarchical structures within communities (Audi, 2020), potentially making religious individuals more susceptible to corruption. Gaan (2014) emphasizes that religious conflicts in India, particularly between Christians and Hindus, can trigger societal unrest and foster corruption. Additionally, such tensions may foster mistrust among communities in diverse settings (Eskridge, 2005). This distrust and violence can also occur within the same faith; for example, Indonesia faces conflicts over different interpretations of Islam, rather than a secular versus religious divide, which sometimes results in violent acts organized by family and study groups (Sheikh, 2019). Conversely, when countries like Malaysia manage social tensions through political or legal means rather than widespread physical conflicts, the adverse effects of religious tensions are likely to decrease (Noor, 2019).

Countries experiencing high religious tension might face a greater risk of corruption among religious individuals. Religious tension reflects religious groups’ efforts to gain power, which can drive religious people to engage in corrupt activities to gain influence. This situation differs significantly from that in countries with low levels of religious tension; thus, we will rerun our regression, excluding countries with high levels of religious tension, using the ICRG data. These countries are Iran, Iraq, Indonesia, Pakistan, and Nigeria. The countries included in our sample, as tested in Table 5, are Bangladesh, Libya, Malaysia, Tunisia, and Türkiye.

**Table 5:** The role of religiosity and criminality on tolerance for corruption, excluding high religious tension countries.

Dependent variable	[1]	[2]	[3]	[4]	[5]
Claiming an unentitled government benefit					
Religiosity	0.223***	0.014	0.023	−0.020	0.129**
	**(3.63)**	(0.66)	(0.21)	(−0.48)	**(2.30)**
Amoral familism	−0.014	−0.099	−0.106	−0.115	−0.086
	(−0.07)	(−0.49)	(−0.53)	(−0.58)	(−0.43)
Anomie theory	0.030*	0.027	0.026	0.026	0.027
	**(1.78)**	(1.61)	(1.56)	(1.55)	(1.62)
Broken window	0.527***	0.529***	0.528***	0.528***	0.525***
	**(24.23)**	**(24.25)**	**(24.25)**	**(24.23)**	**(24.10)**
Rational choice	−0.040***	−0.038***	−0.037***	−0.038***	−0.038***
	**(−2.56)**	**(−2.40)**	**(−2.39)**	**(−2.40)**	**(−2.40)**
Cheating on taxes					
Religiosity	−0.071	−0.080***	0.176	0.004	0.042
	(−1.02)	**(−3.48)**	(1.39)	(0.08)	(0.62)
Amoral familism	0.411**	0.374**	0.482***	0.442**	0.449***
	**(2.14)**	**(1.96)**	**(2.51)**	**(2.33)**	**(2.36)**
Anomie theory	0.031	0.026	0.033*	0.032	0.032
	(1.55)	(1.32)	**(1.66)**	(1.62)	(1.63)
Broken window	0.677***	0.672***	0.677***	0.676***	0.675***
	**(25.29)**	**(25.17)**	**(25.28)**	**(25.26)**	**(25.20)**
Rational choice	−0.020	−0.021	−0.019	−0.021	−0.020
	(−1.04)	(−1.09)	(−1.03)	(−1.09)	(−1.07)
Accepting bribes					
Religiosity	−0.268***	−0.107***	0.165	−0.060	0.140**
	**(−3.75)**	**(−4.30)**	(1.19)	(−1.08)	**(2.00)**
Amoral familism	0.420**	0.448**	0.572***	0.531**	0.560***
	**(2.05)**	**(2.23)**	**(2.77)**	**(2.61)**	**(2.74)**
Anomie theory	0.050**	0.046**	0.055***	0.054***	0.054***
	**(2.30)**	**(2.16)**	**(2.57)**	**(2.54)**	**(2.55)**
Broken window	0.605***	0.597***	0.599***	0.599***	0.596***
	**(22.82)**	**(22.64)**	**(22.66)**	**(22.66)**	**(22.49)**
Rational choice	−0.051***	−0.052***	−0.052***	−0.052***	−0.051***
	**(−2.43)**	**(−2.50)**	**(−2.48)**	**(−2.52)**	**(−2.46)**

Models 1, 2, 3, 4, and 5 represent conceptual, practical, nominal, obedient, and affiliated religiosity, respectively. *, **, and *** indicate statistically significant 10%, 5%, and 1%, respectively. Z-statistics are in the parentheses. Bold numbers indicate statistically significant coefficients, with p-values at 1%, 5%, or 10%.

Coefficients on our main variables of religiosity and criminality were largely significant and had the expected signs, except for the coefficient on conceptual religiosity for the dependent variable “claiming an unentitled government benefit” and the coefficient on affiliated religiosity for the dependent variable “accepting bribes.” Criminality indices were highly significant and had the expected sign, particularly for “broken windows” and “rational choice,” consistent with our main results in Tables 2–4. These two criminology theories explaining individuals’ attitudes toward corruption appear to be more robust than religiosity, regardless of a country’s level of religious tension.

#### 4.2.2 Religiosity and corruption in the least religious respondents

It would be better to test the validity of religion’s effects on tolerance for corruption by re-running our regression specifications on non-Muslim or atheist respondents, where the influence of religiosity indicators on tolerance for corruption should diminish or disappear. However, since our data includes only Muslims in Muslim-majority countries, we can only retest by excluding highly religious respondents. This approach aims to determine whether the religiosity coefficients weaken or vanish, increasing our confidence that the previously significant results were genuinely related to religiosity rather than chance.

Table 6 indicates that the results for religious coefficients, excluding those with high revealed religiosity, diminish or become insignificant across most models using alternative corruption measures. In contrast, the measures of criminality mostly stay significant and retain their original signs. Specifically, the coefficients for obedient religiosity are less significant than in earlier results from models that measure tolerance for corruption via unentitled government benefits and bribe acceptance. While conceptual and practical religiosity appear significant in our main findings (Tables 3, 4), they become insignificant in our restricted sample (Table 6). Only three coefficients across all models and specifications remain significant, compared to the main results for the full sample. Overall, the significance of religiosity coefficients tends to disappear when the sample is limited to the least religious, whereas criminology coefficients largely remain significant, indicating that criminology explains attitudes toward corruption regardless of religiosity.

**Table 6:** The role of religiosity and criminality on tolerance for corruption, excluding religious respondents.

Dependent variable	[1]	[2]	[3]	[4]	[5]
Claiming an unentitled government benefit					
Religiosity	0.326	−0.060	−0.431	0.244	0.218***
	(1.21)	(−0.75)	(−1.42)	(1.23)	**(2.74)**
Amoral familism	−0.514*	−0.426	−0.108	−0.250	−0.067
	**(−1.68)**	(−1.59)	(−0.47)	(−1.25)	(−0.54)
Anomie theory	0.028	0.007	0.027	0.063***	0.039***
	(0.73)	(0.17)	(0.94)	**(2.46)**	**(3.24)**
Broken window	0.574***	0.457***	0.348***	0.531***	0.507***
	**(13.42)**	**(10.07)**	**(10.91)**	**(16.35)**	**(30.91)**
Rational choice	−0.043	−0.057*	−0.012	−0.044*	−0.029***
	(−1.27)	**(−1.66)**	(−0.50)	**(−1.77)**	**(−2.48)**
Cheating on taxes					
Religiosity	−0.176	0.065	−0.601*	−0.069	0.070
	(−0.62)	(0.76)	**(−1.77)**	(−0.30)	(0.77)
Amoral familism	−0.042	−0.163	0.409*	−0.068	0.145
	(−0.14)	(−0.60)	**(1.72)**	(0.31)	(1.12)
Anomie theory	0.007	0.064	−0.043	0.013	0.001
	(0.18)	(1.48)	(−1.21)	(0.44)	(0.05)
Broken window	0.584***	0.764***	0.584***	0.686***	0.629***
	**(12.81)**	**(13.18)**	**(14.03)**	**(17.48)**	**(32.44)**
Rational choice	−0.054	−0.037	−0.049	−0.015	−0.050***
	(−1.45)	(−0.97)	(−1.63)	(−0.51)	**(−3.67)**
Accepting bribes					
Religiosity	−0.217	−0.072	−0.542	0.175	0.194*
	(−0.74)	(−0.78)	(−1.54)	(0.70)	**(1.99)**
Amoral familism	−0.153	0.589**	0.746***	−0.284	0.143
	(−0.44)	**(2.33)**	**(3.14)**	(−1.10)	(0.99)
Anomie theory	0.009	0.092*	0.021	0.045	0.018
	(0.20)	**(1.88)**	(0.60)	(1.39)	(1.19)
Broken window	0.455***	0.555***	0.378***	0.504***	0.499***
	**(10.38)**	**(9.91)**	**(10.06)**	**(14.15)**	**(26.94)**
Rational choice	−0.054	−0.138***	−0.012	−0.034	−0.056***
	(−1.32)	**(−3.17)**	(−0.39)	(−1.07)	**(−3.80)**

Models 1, 2, 3, 4, and 5 represent conceptual, practical, nominal, obedient, and affiliated religiosity, respectively. *, **, and *** indicate statistically significant 10%, 5%, and 1%, respectively. Z-statistics are in the parentheses. Bold numbers indicate statistically significant coefficients, with p-values at 1%, 5%, or 10%.

#### 4.2.3 Religiosity and corruption using an ordered probit model

Although ordered probit and ordered logit are very similar near the middle of their cumulative distribution curves, they differ in their assumptions about the error term’s distribution. Ordered logit may be more suitable since its results can be interpreted as odds ratios and it handles outliers well. However, ordered probit’s assumption of normally distributed errors aligns naturally with the underlying latent variable approach. Thus, Table 7 presents the same model specifications as in Tables 2–4, but excludes country- and individual-level control variables due to space limitations.

**Table 7:** The role of religiosity and criminality on tolerance for corruption, using an ordered probit model.

Dependent variable	[1]	[2]	[3]	[4]	[5]
Claiming an unentitled government benefit					
Religiosity	0.025	0.002	−0.059	−0.050***	0.027
	(1.13)	(0.25)	(−1.37)	**(−3.06)**	(1.36)
Amoral familism	−0.128*	−0.132**	−0.139**	−0.144**	−0.129*
	**(−1.86)**	**(−1.92)**	**(−2.02)**	**(−2.10)**	**(−1.88)**
Anomie theory	0.020***	0.019***	0.019***	0.019***	0.019***
	**(3.23)**	**(3.18)**	**(3.17)**	**(3.14)**	**(3.20)**
Broken window	0.238***	0.238***	0.237***	0.237***	0.237***
	**(32.12)**	**(32.08)**	**(32.04)**	**(32.02)**	**(31.96)**
Rational choice	−0.019***	−0.019***	−0.019***	−0.018***	−0.020***
	**(−3.29)**	**(−3.22)**	**(−3.23)**	**(−3.13)**	**(−3.26)**
Cheating on taxes					
Religiosity	−0.066***	−0.049***	0.041	−0.011	−0.020
	**(−2.70)**	**(−4.79)**	(0.87)	(−0.65)	(−0.92)
Amoral familism	0.054	0.039	0.072	0.066	0.065
	(0.77)	(0.54)	(1.02)	(0.94)	(0.92)
Anomie theory	−0.003	−0.003	−0.002	−0.002	−0.003
	(−0.55)	(−0.58)	(−0.37)	(−0.38)	(−0.40)
Broken window	0.286***	0.286***	0.287***	0.286***	0.287***
	**(36.43)**	**(36.36)**	**(36.48)**	**(36.44)**	**(36.45)**
Rational choice	−0.027***	−0.028***	−0.028***	−0.028***	−0.028***
	**(−4.06)**	**(−4.20)**	**(−4.26)**	**(−4.24)**	**(−4.23)**
Accepting bribes					
Religiosity	−0.070***	−0.044***	−0.062	−0.041**	0.009
	**(−2.71)**	**(−4.09)**	(−1.27)	**(−2.16)**	(0.41)
Amoral familism	0.017	0.00008	0.026	0.024	0.034
	(0.22)	(0.00)	(0.34)	(0.32)	(0.45)
Anomie theory	0.004	0.005	0.005	0.005	0.006
	(0.69)	(0.65)	(0.81)	(0.83)	(0.84)
Broken window	0.242***	0.241***	0.242***	0.242***	0.242***
	**(30.68)**	**(30.62)**	**(30.68)**	**(30.66)**	**(30.63)**
Rational choice	−0.026***	−0.027***	−0.028***	−0.027***	−0.028***
	**(−3.71)**	**(−3.83)**	**(−3.89)**	**(−3.83)**	**(−3.91)**

Models 1, 2, 3, 4, and 5 represent conceptual, practical, nominal, obedient, and affiliated religiosity, respectively. *, **, and *** indicate statistically significant 10%, 5%, and 1%, respectively. Z-statistics are in parentheses. Bold numbers indicate statistically significant coefficients, with p-values at 1%, 5%, or 10%.

Results shown in Table 7 closely match those of ordered logit models in terms of the direction and significance of religiosity and criminological coefficients. However, the effect sizes in ordered probit models are smaller than in ordered logit models. Notably, obedient religiosity typically decreases Muslims’ likelihood of justifying unentitled government benefits or accepting bribes. Both conceptual and practical religiosity are generally associated with Muslims being less tolerant of cheating on taxes or accepting bribes. Regarding criminological indices, the broken windows and rational choice measures consistently and significantly predict Muslims’ attitudes toward corruption across all models and alternative corruption indicators.

## 5 Discussion

This study examines how religiosity shapes Muslims’ attitudes toward corruption. The main findings indicate that higher religiosity among Muslims is generally associated with lower tolerance for corruption, as shown in Tables 2–4 However, the significance varies by the type of religiosity and the corruption measure used. Additionally, our results are not definitive, as some coefficients were not significant, consistent with previous research (Valdovinos-Hernandez et al., 2019; Ko and Moon, 2014).

Both conceptual and practical religiosity are notably associated with less tolerance for accepting bribes and cheating on taxes. However, these forms of religiosity do not significantly associate with tolerance for claiming unentitled government benefits. Obedient religiosity is associated with Muslims being less tolerant of accepting bribes and of claiming unentitled benefits, but it does not substantially contribute to tax evasion. Meanwhile, affiliated religiosity does not appear to be significantly associated with any of the main models. These results hold even when we introduce alternative estimates from ordered probit models.

Criminological indices showed greater consistency and predictive power for Muslims’ tolerance of corruption, particularly when broken windows and rational choice indices were examined. These results align with existing research indicating that religion can contribute to less corruption when institutions are strong (Sommer et al., 2012). All models that include these indices significantly predict Muslims’ attitudes toward claiming unentitled government benefits, cheating on taxes, and accepting bribes. The broken window theory highlights the influence of a culture of unspoken violations that increases individuals’ justification of corrupt acts (Keuschnigg and Wolbring, 2015; Schram et al., 2022), while the rational choice theory emphasizes how institutions and the rule of law shape attitudes toward corruption (Graycar and Sidebottom, 2012; Zimring and Johnson, 2005; Suparman et al., 2024). Notably, the potential consequences of accountability appear to be more strongly associated with individuals’ anti-corruption attitudes than with their religiosity. These results are supported by odds ratios (reported in Appendix Table 9) and the main analysis tables; however, they suggest that criminological indices are more explanatory of respondents’ attitudes toward corruption than religiosity.

Criminological and institutional factors might have a stronger influence because they interact directly with individuals. Moreover, Islamic rules tend to focus on rewards or punishments in the afterlife, contrasting with institutional and criminal systems where punishments are immediate upon detecting corruption. It’s also worth noting that our sampled countries include nations with Muslim majorities where Islamic law is not actively enforced in corruption issues. If enforcement were stricter, it might imply a connection between Islamic law and anti-corruption attitudes. However, the application of Sharia law varies across these countries, often limited to family and personal status matters rather than anti-corruption laws.

This study challenges the common view that religion is positively linked to corruption, not only through its findings but also in its methodology. Unlike other research, we do not distinguish between hierarchical and non-hierarchical religions (Xu et al., 2017; Zelekha and Avnimelech, 2023; Paldam, 2001); instead, we assess religiosity at the individual level. Our approach avoids macro-level analysis (Gokcekus and Ekici, 2020; Zelekha and Avnimelech, 2023; Ali, 2025), although we do consider differences by individuals’ country of residence. Additionally, we do not limit religiosity to a single perspective (Valdovinos-Hernandez et al., 2019; Ali, 2025), nor do we rely on expert opinions on corruption (Valdovinos-Hernandez et al., 2019). Instead, we use various measures of religiosity and link them to individuals’ tolerance for corruption. This approach is more effective at capturing the association between religiosity and tolerance for corruption while accounting for other criminological factors.

## 6 Conclusion

This research examines the link between religiosity and tolerance for corruption in predominantly Muslim countries. It highlights a contradiction in some studies, which show that although Islamic teachings are believed to discourage corruption, empirical evidence often yields inconsistent results on the relationship between religiosity and corruption. Nonetheless, criminological theories indicate that, regardless of one’s religion, certain factors influence individuals’ attitudes toward corruption.

The results show that religiosity, when statistically significant, is positively associated with respondents’ attitudes toward anti-corruption, though fewer coefficients reach statistical significance. Nonetheless, religiosity-related variables have limited explanatory power compared with criminological theories. Criminological theories such as the rational choice and broken windows models are more effective, emphasizing perceived detection risk and tolerance of minor violations as key factors in shaping attitudes toward corruption. Furthermore, religious teachings against corruption might encourage more devout individuals to be less tolerant of it. However, simply attributing this to anti-corruption Islamic teachings and expecting it to manifest more among religious people may not be sufficient to establish the underlying mechanism.

The findings suggest that addressing tolerance of minor offenses through enhanced law enforcement, stronger institutional accountability, and improved governance is more effective in fostering anti-corruption attitudes than relying solely on moral or religious appeals. Additionally, increased accountability can raise the costs and risks of corruption, potentially increasing the criminological risk for individuals and encouraging stronger institutional standards. These combined measures may contribute to stronger anti-corruption attitudes in Muslim-majority societies. Nonetheless, we would like to highlight several limitations of our findings: the WVS captures attitudes, not actual behavior; the cross-sectional design limits causal conclusions; the analysis is limited to Muslim-majority countries; the role of criminality factors appears to stem from the varied and mixed legal systems across our country sample; and religiosity is measured through self-reported responses. Finally, removing “No answer,” “Not applicable,” or “Do not know” responses, along with other rows to standardize respondents across countries, presumes that missing data are random. However, these options are a limitation that may introduce bias into our final sample.

In conclusion, a deeper understanding of the factors that shape Muslims’ tolerance for corruption is necessary. These factors may include deficiencies in religiosity, morality, or institutional effectiveness in Muslim societies. Our research offers strong evidence that strengthening institutions and law enforcement can substantially contribute to explaining why Muslims are less tolerant of corruption. Additionally, higher levels of religiosity are associated with lower tolerance for corruption among Muslims.

## Data Availability

Publicly available datasets were analyzed in this study. This data can be found at: https://www.worldvaluessurvey.org/WVSDocumentationWV7.jsp.

## Footnotes

1 Transparency International (TI) (2024) reports that anti-corruption initiatives in the Middle East and North Africa (mainly Muslim countries) remain weak. The Corruption Perception Index (CPI) score has risen just one point over 12 years, reaching 39 out of 100, which is among the lowest regional averages.

2 Obedience is calculated as (Q8 + Q14) – (Q15 + Q17). Since these questions are binary—where 1 indicates a valued trait, and 2 indicates a trait not valued—the obedience score can range from 2 to −2. A higher score signifies a greater promotion of obedience at home.

3 Conceptual, practical, and nominal religiosity were rescaled so that higher values refer to higher religiosity.
