# Supplementary material for: Religiosity and corruption tolerance in Muslim-majority countries
Source: Front Sociol. 2026 Sep 3;11:1858920. doi: 10.3389/fsoc.2026.1858920 (PMC13581831; doi:10.3389/fsoc.2026.1858920)
Supplement: Supplementary file 1 [file Supplementary_file_1.docx]

**Appendix**

**Country list**

Bangladesh, Indonesia, Iran, Iraq, Libya, Malaysia, Nigeria, Pakistan, Tunisia, Türkiye.

**Table VIII.** Muslim population (%) and legal system implemented.

| **Country** | **% of Muslims** | **Legal system** | **Mixed system components** |
| --- | --- | --- | --- |
| **Bangladesh** | 90.80% | Mixed | Muslim/ Common law |
| **Indonesia** | 87.00% | Mixed | Civil law/Muslim/Customary |
| **Iran** | 99.50% | Mixed | Muslim/Civil law |
| **Iraq** | 99.10% | Mixed | Muslim/Civil law |
| **Libya** | 96.60% | Mixed | Muslim/Civil law |
| **Malaysia** | 66.10% | Mixed | Muslim/Common law/Customary |
| **Nigeria** | 51.10% | Mixed | Muslim/Common law/Customary |
| **Pakistan** | 96.5% | Mixed | Muslim/Common law |
| **Tunisia** | 99.50% | Mixed | Civil law/Muslim |
| **Turkey** | 98.00% | Civil law | -- |

**Source:** For Muslim population: <https://worldpopulationreview.com/country-rankings/muslim-majority-countries>, for legal system: <https://www.juriglobe.ca/eng/sys-juri/index-alpha.php>.

**Table IX.** Odds ratio estimate for main coefficients in Table II, III, & IV

|  | **Table II** | | | | |
| --- | --- | --- | --- | --- | --- |
| **Variables** | **[1]** | **[2]** | **[3]** | **[4]** | **[5]** |
| **Religiosity indices** | 0.981 | 0.999 | 0.936 | **0.909** | 1.030 |
| **Amoral familism** | 0.840 | 0.836 | 0.831 | **0.819** | 0.841 |
| **Anomie theory** | **1.033** | **1.033** | **1.033** | **1.032** | **1.033** |
| **Broken Window** | **1.590** | **1.590** | **1.588** | **1.588** | **1.588** |
| **Rational choice** | **0.964** | **0.964** | **0.964** | **0.965** | **0.964** |
|  | **Table III** | | | | |
| **Variables** | **[1]** | **[2]** | **[3]** | **[4]** | **[5]** |
| **Religiosity indices** | **0.911** | **0.913** | 1.055 | 0.957 | 0.951 |
| **Amoral familism** | 1.091 | 1.060 | 1.120 | 1.106 | 1.107 |
| **Anomie theory** | 0.999 | 0.998 | 1 | 1 | 1 |
| **Broken Window** | **1.759** | **1.755** | **1.762** | **1.761** | **1.762** |
| **Rational choice** | **0.951** | **0.950** | **0.950** | **0.950** | **0.950** |
|  | **Table IV** | | | | |
| **Variables** | **[1]** | **[2]** | **[3]** | **[4]** | **[5]** |
| **Religiosity indices** | **0.889** | **0.921** | 0.912 | **0.915** | 0.992 |
| **Amoral familism** | 1.036 | 1.012 | 1.057 | 1.049 | 1.063 |
| **Anomie theory** | 1.009 | 1.008 | 1.011 | 1.011 | 1.011 |
| **Broken Window** | **1.562** | **1.560** | **1.562** | **1.562** | **1.563** |
| **Rational choice** | **0.950** | **0.949** | **0.948** | **0.949** | **0.948** |

**Note:** Religiosity indices refer to conceptual, practical, nominal, and affiliated religiosity, respectively. Odds ratios appeared in bold if the corresponding coefficient is significant.

| **Table X.** The threshold (Limit Points) for Tables II, III, and IV | | | | | |
| --- | --- | --- | --- | --- | --- |
| **Threshold (Limit Points) of regression models in Table II** | | | | | |
| **Limit points** | **[1]** | **[2]** | **[3]** | **[4]** | **[5]** |
| **Limit 1 (Score 1/ Score 2)** | 1.626 | 1.650 | 1.721 | 1.519 | 1.686 |
| **Limit 2 (Score 2/ Score 3)** | 2.165 | 2.189 | 2.260 | 2.060 | 2.225 |
| **Limit 3 (Score 3/ Score 4)** | 2.618 | 2.642 | 2.713 | 2.513 | 2.678 |
| **Limit 4 (Score 4/ Score 5)** | 2.984 | 3.008 | 3.079 | 2.880 | 3.045 |
| **Limit 5 (Score 5/ Score 6)** | 3.599 | 3.623 | 3.694 | 3.495 | 3.659 |
| **Limit 6 (Score 6/ Score 7)** | 4.046 | 4.069 | 4.140 | 3.942 | 4.106 |
| **Limit 7 (Score 7/ Score 8)** | 4.393 | 4.417 | 4.487 | 4.290 | 4.454 |
| **Limit 8 (Score 8/ Score 9)** | 4.830 | 4.853 | 4.924 | 4.726 | 4.890 |
| **Limit 9 (Score 9/ Score 10)** | 5.235 | 5.258 | 5.329 | 5.132 | 5.295 |
| **Threshold (Limit Points) of regression models in Table III** | | | | | |
| **Limit points** | **[1]** | **[2]** | **[3]** | **[4]** | **[5]** |
| **Limit 1 (Score 1/ Score 2)** | 1.844 | 1.895 | 1.681 | 1.682 | 1.681 |
| **Limit 2 (Score 2/ Score 3)** | 2.555 | 2.609 | 2.392 | 2.393 | 2.392 |
| **Limit 3 (Score 3/ Score 4)** | 3.163 | 3.219 | 3.001 | 3.001 | 3.001 |
| **Limit 4 (Score 4/ Score 5)** | 3.641 | 3.698 | 3.478 | 3.479 | 3.479 |
| **Limit 5 (Score 5/ Score 6)** | 4.353 | 4.410 | 4.189 | 4.191 | 4.190 |
| **Limit 6 (Score 6/ Score 7)** | 4.937 | 4.993 | 4.773 | 4.775 | 4.775 |
| **Limit 7 (Score 7/ Score 8)** | 5.414 | 5.471 | 5.250 | 5.252 | 5.252 |
| **Limit 8 (Score 8/ Score 9)** | 6.047 | 6.105 | 5.883 | 5.886 | 5.885 |
| **Limit 9 (Score 9/ Score 10)** | 6.388 | 6.447 | 6.225 | 6.228 | 6.227 |
| **Threshold (Limit Points) of regression models in Table IV** | | | | | |
| **Limit points** | **[1]** | **[2]** | **[3]** | **[4]** | **[5]** |
| **Limit 1 (Score 1/ Score 2)** | 2.026 | 2.042 | 2.008 | 1.785 | 1.892 |
| **Limit 2 (Score 2/ Score 3)** | 2.753 | 2.770 | 2.733 | 2.511 | 2.618 |
| **Limit 3 (Score 3/ Score 4)** | 3.370 | 3.389 | 3.351 | 3.129 | 3.236 |
| **Limit 4 (Score 4/ Score 5)** | 3.764 | 3.784 | 3.746 | 3.524 | 3.630 |
| **Limit 5 (Score 5/ Score 6)** | 4.269 | 4.289 | 4.249 | 4.029 | 4.134 |
| **Limit 6 (Score 6/ Score 7)** | 4.734 | 4.754 | 4.713 | 4.495 | 4.598 |
| **Limit 7 (Score 7/ Score 8)** | 5.126 | 5.146 | 5.106 | 4.889 | 4.991 |
| **Limit 8 (Score 8/ Score 9)** | 5.729 | 5.749 | 5.710 | 5.493 | 5.595 |
| **Limit 9 (Score 9/ Score 10)** | 6.261 | 6.282 | 6.244 | 6.027 | 6.128 |
